# Systemic and Oral Immunogenicity of Porcine Epidemic Diarrhea Virus Antigen Fused to Poly-Fc of Immunoglobulin G and Expressed in ΔXT/FT *Nicotiana benthamiana* Plants

**DOI:** 10.3389/fphar.2021.653064

**Published:** 2021-04-30

**Authors:** Nguyen-Quang-Duc Tien, Moon-Sik Yang, Yong-Suk Jang, Tae-Ho Kwon, Rajko Reljic, Mi-young Kim

**Affiliations:** ^1^ Department of Molecular Biology, Jeonbuk National University, Jeonju-Si, South Korea; ^2^ NBM Inc., Wanjusandan 5-ro, Bongdong-eup, Wanju-Gun, Jeollabuk-do, South Korea; ^3^ University of Sciences, Hue University, Hue City, Viet Nam; ^4^ Institute for Infection and Immunity, St George’s University of London, London, UK

**Keywords:** vaccine, PEDV, mucosal, immunity, plant

## Abstract

Porcine epidemic diarrhea virus (PEDV), a member of the Coronaviridae family has become increasingly probelmatic in the pig farming industry. Currently, there are no effective, globally applicable vaccines against PEDV. Here, we tested a recombinant PEDV vaccine candidate based on the expression of the core neutralising epitope (COE) of PEDV conjugated to polymeric immunoglobulin G scaffold (PIGS) in glycoengineered *Nicotiana be* *nthamiana* plants. The biological activity of COE-PIGS was demonstrated by binding to C1q component of the complement system, as well as the surface of antigen-presenting cells (APCs) *in vitro.* The recombinant COE-PIGS induced humoral and cellular immune responses specific for PEDV after both systemic and mucosal vaccination. Altogether, the data indicated that PEDV antigen fusion to poly-Fc could be a promising vaccine platform against respiratory PEDV infection.

## Introduction

Pork is the most widely eaten meat in the world; approximately, 1.3 billion pigs are produced each year worldwide. The intensification and globalization of the swine industry has contributed to the emergence and global spread of pathogens of swine, driven in part by frequent movements of pigs, feed, and pork products at local, national, and international scales (Drew, 2011). For example, porcine epidemic diarrhea (PED) spread from China to the United States (discovered in 2013) and thereafter to Canada and Mexico (in 2014) (Lee, 2015). Large-scale PED epidemics recurred through the pork industry and within 1 year, the virus had impacted ∼50% of US breeding herds, resulting in the deaths of at least seven million piglets (Ojkic et al., 2015; Goede and Morrison, 2016). Similarly, African swine fever (ASF) emerged in Eastern Europe from sub-Saharan Africa in 2007 and recently re-emerged in most of Asia including China, Philippines, Vietnam and East Timor in 2018–2019 (Blome et al., 2020). There are risks of the virus spreading also to other countries, as shown by its recent 2019 introduction to China (the world’s largest producer of pigs) and spread to neighboring countries such as North and South Korea. Cases in South Korea were speculated to have occurred by pigs crossing the demilitarized zone which is a 4 km-wide strip of land that is a buffer zone between North and South Korea. Despite extensive precautions following first recorded cases in North Korea in May 2019, South Korea reported its first case in September 2019 and has culled about 400,000 pigs after an outbreak began late last year (https://www.thepigsite.com/news/2020/10/south-korean-officials-cull-1-500-pigs-after-asf-outbreak). More than six million pigs have been culled overall in Asia (https://www.bbc.co.uk/news/world-asia-49916065). Finally, the potential importance of livestock pathogens for human public health was exemplified by the H1N1 “swine flu” pandemic in 2009, which originated from influenza A viruses circulating in pig populations (Smith et al., 2009). These examples highlight the need to build a global picture of pathogens of swine to enhance preparedness and understand patterns of emergence and spread. Thus, a versatile and efficient vaccine platform for swine diseases would be helpful to easily and quickly develop new vaccines against unknown or new pathogens which show a similar infection pathway.

Recently, PED caused by coronavirus infection has re-emerged, resulting in a substantial economic burden in pig industry. The first recorded case was in the United Kingdom in 1971 (Antas and Wozniakowski, 2019) and it subsequently spread to many European countries, followed by the current epidemic in Asian countries. Increasing problem is due to an appearance of new variants of PEDV with deletions/insertion, and several mutations in S (spike) 1 region (Lee et al., 2010; Lee, 2015; Jang et al., 2019). The reason for partial protection against PEDV with the current vaccine based on the CV777 strain could be explained by several sequence analyses. Zhao X. et al. have compared the sequences between their 55 isolated Chinese field strains (during 2011–2015) and foreign reference strains from GenBank. The S genes shared identity with CV777 (93.8–95.7%), CHGD-01 (94.6.0–98.3%), M98 (93.3–95.4%), attenuated DR13 (93.3–95.6%), and 83P-5_100th-passaged (93.7–96.0%) and shared nucleotide (deduced amino acid) homologies of 96.1–99.2% (95.1–99.5%) with United States/Missouri102/2013 (Zhao et al., 2017). New isolates from Korean PEDV epidemic strain, PED-CUP-B2014, were close to PEDVs currently circulating in many countries including the United States, and are distinct from many current vaccine strains (Park et al., 2018). We also compared the sequences of S protein between CV777 and various strains provided by NCBI including Korean (AAM19716.1), Chinese (AGN29320.1) and Vietnamese (ASU09581.1) isolates and the homology was 94, 95.9, 92.1 and 91.9%, respectively (Table 1).

**TABLE 1:** Summary of S protein of PEDV published in NCBI and UniProt.

GeneBank/UniProtKB	S* (aa)	Homology (%) vs. CV777	S1	S2	Fusion peptide	Coiled coil	TM	Motif (KxHxx)	Interaction with host receptor (ANPEP) and note
UniRule: UR000865645	16–1,173		16–536	537–1,173	753–773	1,063–1,105 834–878		1,169–1,173	417–547
	Belongs to the alphacoronaviruses spike protein family. The KxHxx motif seems to function as an ER retrieval signal. In contrast to sergoroups 2 and 3, glycoprotein from seogroup 1 is not cleaved into S and S2. Homotrimer. During virus morpphogenesis, found in a complex with M and HE proteins. Interacts with host ANPEP. Note: Accumulates in the endoplasmic reticulum-Golgi intermediate compartment, where it participates in virus particle assembly.								
AAK38656.1	1,383	-	231–733	741–1,344					CV77, Pensaert and De Bouck (1978)
A0A5B8YRH6	1,386	9.2% identity (97.0% similar)	234–736	744–1,385	958–978	1,039–1,083 1,275–1,317	1,328–1,347	1,382–1,386	100% identity to AAk3865631 in Uniprot Blast
AAM19716.1	1,386	92.5% identity (97.0% similar)	234–736	744–1,347					Korean isolate; Kang et al. (2005)
Q8QQ98	1,386	92.5% identity (97.0% similar)	234–736	744–1,385	958–978	1,039–1,083 1,275–1,317	1,328–1,347	1,382–1,386	100% identity to AAM19716.1 in Uniprot Blast
AGN293201	1,382	95.9% identity (98.4% similar)	230–732	740–1,343					a new Porcineepidemic diarrhea virus isolated of China; Chen et al. (2012)
R9TI84	1,382	95.9% identity (98.4% similar)	230–732	740–1,381	954–974	1,035–1,079 1,271–1,313	1,324–1,343	1,378–1,382	100% identity to AGN29320.1 in Uniprot Blast
AST13221.1	1,380	97.8% identity (99.3% similar)	235–737	745–1,348					85–7 mutant strain 1; Sun et al. (2017)
A0A288Y8U4	1,380	98.1% identity (99.3% similar)	235–737	745–1,378	959–979	1,040–1,084 1,276–1,318	1,329–1,348		100% identity to AST13221.1 in Uniprot Blast
ASU09581.1	1,386	91.9% identity (96.6% similar)	234–736	744–1,347					HUA–PED96 Vietnam; Do Hai et al. (2016)
A0AU2UB55	1,386	91.9% identity (96.6% similar)	234–736	744–1,385	958–978	1,039–1,083 1,275–1,317	1,328–1,347	1,382–1,386	100% identity to ASU09581.1 Uniprot Blast
MK841495 (QED40665.1)	1,386	92.7% identity (97.0% similar)							PEDV YZ, China; Wang et.al. (2019)
A0A5B8YRH6	1,386	92.7% identity (97.0% similar)	234–736	744–1,385	958–978	1,039–1,083 1,275–1,317	1,328–1,347	1,382–1,386	100% identity to MK841495 in Uniprot Blast

* Spike (S) protein consists with two domains (S1 and S2) with transmembrane (TM). The residues corresponding to each protein positions were represented in the table. Homology (%) is compared to corona-like agent (CV777) as the causative pathogan, which was partially fulfilled Koch’s postulates and described by Belgium scientists at the Ghent University in 1978. The Annotation and conditions in the UniRule (UR000865645) are derived from the following entries; P15423 (SPIKE_CVH22).

Over the past several years, platform technologies have been developed that could make it possible for multiple vaccines to be rapidly produced from a single system. Here we suggest molecularly engineered adjuvant, named PIGS (Polymeric Immunoglobulin Scaffold), which we demonstrated previously to have an excellent *in vitro*, *in vivo* and *ex vivo* adjuvantic properties (Kim et al., 2017b; Kim et al., 2018; Webster et al., 2018). Thus, the vaccine comprises the antigen of choice genetically linked to the poly-Fc domain of modified IgG containing IgM-polymerisation C-terminal sequence, enabling its polymerization similar to pentameric IgM. This is the basis of its enhanced binding to complement and Fc gamma immunoglobulin receptors. We tested this concept for induction of systemic immunity against dengue infection in mice (Kim et al., 2018). For a swine PEDV vaccine, PIGS fused to PEDV antigen were tested by systemic and mucosal administration. Mucosal (oral) immunization would be highly desirable against the respiratory and enteric diseases caused by complex bacterial and viral agents, as the induced responses could potentially stop the infection before it can spread.

In this study, COE epitope antigen of the S1 protein of PEDV, which is responsible for inducing neutralizing antibodies (Chang et al., 2002; Chang et al., 2019; Ho et al., 2020; Sun et al., 2008), was used to test the poly-Fc based vaccine against swine infection. The COE and PIGS fusion protein were transiently expressed in *Nicotiana benthamiana* plants and both systemic and oral immunogenicity were analyzed in the mouse model.

## Materials and Methods

### Gene Construction for Plant Expression Vector

The core neutralizing epitope (COE) composed of 139 amino acid (aa) length (aa position 499 to 637 of PEDV strain CV777 S gene; UniProtKB-Q91AV1) was selected to develop PEDV vaccine and synthesized with the plant codon optimization sequences. To verify enhanced immunogenicity, an antigen delivery molecule, a mouse IgG2a Fc based polymeric IgG scaffold (mPIGS) has been fused to COE, which had demonstrated an increased antigen specific antibody response and self-adjuvancity in our previous dengue vaccine study (Kim et al., 2017b). The mPIGS gene fragment was cloned into pGEM T-easy vector (Promega, United States) by PCR amplification method using the plasmid pMPIGS as the DNA template, with gene-specific primers 5′-gcg​gta​ccG​CTT​CAT​CTA​CAA​AA-3′ (forward) and 5′-gc gag​ctc​TTA​GTA​GCA​AGT​GCC-3′ (reverse). The PCR conditions were as follows: 30 cycles of PCR amplification (DNA strand denaturation at 94°C for 30 s, annealing at 58°C for 30 s, and complementary strand synthesis at 72°C for 40 s) and a final extension at 72°C for 10 min. The PCR product was confirmed by DNA sequence analysis (COSMO Genetech, South Korea). The gene encoding mPIGS was then fused with COE using KpnI and SacI restriction enzyme sites in the intermediate vector. To construct plant expression vector, the fusion gene (COE-mPIGS, 1,203 kb) was PCR amplified with forward and reverse primers (5′-TTT​TGG​TCT​CA*AGG​T*GCT​TCA​TCT​ACA​AAA-3′; 5′- TTT​TGG​TCT​CA*AAG​C*TTA​GTA​GCA​AGT​GCC-3′, respectively) and then cloned into MagnIcon® transient expression vector, pICH31070 (kindly, provided from Y Gleba, Icon Genetics, Germany). The recombinant plasmid was designated pMYK-T10 and transformed into *Agrobacterium* strain GV3010 using electroporation method (Wise et al., 2006).

### Agroinfiltration for a Transient Expression in Plants

To increase binding avidity to low affinity Fcɤ receptors and CD16a in particular, COE-mPIGS construct was expressed in delta-XF variant tobacco cells which produce only residual xylose and fucose glycans (Kim et al., 2015; Strasser et al., 2008). The ΔXT/FT *N. benthamiana* seeds (kindly, provided from Strasser R., Austria) were germinated in the greenhouse under the conditions of 16 h light/8 h dark cycle at 25 ± 0.5°C. For transient expression of COE-mPIGS protein, pro-vector expression system was employed and *Agrobacterium* cells (strain GV3010) harboring pICH20155 (5′ pro-vector), plasmid pMYK-T10 (3’ pro-vector) and pICH14011 (integrase vector), respectively, were co-infiltrated into 6-weeks old plantlets. All procedures were described in our previous report (Huy and Kim, 2017). Also, LBA404 agrobacterium harboring plasmid encoding J chain, which links together monomeric units in secretory IgA (SIgA) or pentameric IgM in nature (Johansen et al., 2000) was also co-infiltrated, to improve multimerization by forming a disulfide bridge with the mu-tail piece at the C-terminal end of mPIGS (Yoo et al., 1999). Briefly, three agrobacteria (described above) were grown in Luria-Bertani broth (LB) medium containing 50 μg/L kanamycin and 100 μg/L rifampicin for in 100 ml culture at 28°C for 2 days, respectively. The bacterial cells were harvested by centrifugation at 6,000 × g for 5 min and suspensed in 5 L of MES infiltration buffer (10 mM MES, 10 mM MgSO_4_, pH 5.5, 200 µM Acetosyringone). The bacterial suspension solution was adjusted to OD_600_ value = 0.3 and pre-incubated for 2 h at room temperature before agroinfiltration. Vacuum-infiltration was applied to plants immersed in MES infiltration buffer. The bacterial cells penetrated into the leaves by slowly releasing the air pressure after 2 min holding at vacuum 0.1 mPa. The infiltrated plants were further grown in greenhouse under 16 h continuous light per day, and their leaves were harvested at day 6 post infiltration for subsequent experiments.

### Protein Extraction and Purification

To extract COE-mPIGS protein, the infiltrated leaves were homogenised by blender with double volumes of chilled PBS buffer. The crude protein extracts were filtered through two layers of Miracloth (Calbiochem) and centrifuged twice at 14,000 rpm for 30 min in a Beckman centrifuge (Avanti^TM^ J25, United States). The recombinant COE-mPIGS protein in the supernatant was detected by Western blot analysis prior to purification. The supernatant was filtered through 0.22 µm filter before loading onto a protein A agarose affinity column (Sigma) previously equilibrated with the binding buffer. The column was washed with five volumes of washing buffer (20 mM sodium phosphate, pH 7.0). The bound antibodies were eluted with elution buffer (0.1 M glycine-HCl, pH 2.7) and pH immediately neutralized by addition of 1 M Tris base (pH unadjusted). The recombinant protein elution fractions were concentrated by Amicon Ultra- Centrifugal Filter Unit (Millipore, Billerica, MA) and dialyzed using Slide-A-Lyzer Dialysis (Thermo Fisher Scientific) in PBS buffer before quantification by optical density method, at 280 nm. Aliquots of the protein were stored at −20^°^C for further experiments.

### Immunoblot Analysis

An aliquot of protein extracts containing 40 µg of TSP, along with prestained molecular weight markers, were separated by 8% (non-boiled condition) or 10% (boiled condition) SDS-PAGE (Bio-Rad, United States) at 120 V for 2 h in Tris-glycine buffer, pH 8.3 (25 mM Tris, 250 mM glycine and 0.1% SDS). The separated protein bands were transferred onto Hybond C membrane (Amersham Pharmacia Biotech, Piscataway, NJ, United States) in transfer buffer (50 mM Tris, 40 mM glycine, and 20% methanol) using a mini-transblot apparatus (Bio-Rad, United States) at 130 mA for 2 h. To prevent non-specific antibody reactions, membrane was blocked with 10% non-fat milk powder in TBST buffer (Tris-buffered saline with 0.05% Tween 20) with gentle agitation on a rotary shaker at 20 rpm overnight. For detection of mPIGS, the membrane was incubated with anti-mouse heavy chain specific antibodies conjugated to alkaline phosphatase (Sigma A3438, United States) diluted (1:7,000) in TBST buffer containing 0.3% non-fat dry milk. For detection of COE, mouse anti-COE antiserum (diluted 1:5,000) was used. The membrane was then washed three times with TBST buffer and incubated for 2 h with rabbit anti-mouse light chain specific antibodies conjugated with alkaline phosphatase (Sigma SAB3701215, United States), diluted (1:7,000) in TBST buffer. The membranes was washed twice with TBST buffer and once with TMN buffer (100 mM Tris, pH 9.5, 5 mM MgCl_2_, and 100 mM NaCl). The colour was developed using premixed BCIP/NBT solution (Sigma, United States).

### C1q Complement Binding Assay

The first step in activation of the classical complement pathway is the binding of C1q protein to antibodies-antigen complexes. To better define one of the biological functions of COE-mPIGS, we compared the C1q binding activity of these immune complex-like molecules, with monomeric mouse IgG serving as a negative control, by ELISA. The plates were coated with 1 µg/well of human complement C1q (Calbiochem) in 100 µl of coating buffer (15 mM Na_2_CO_3_; 35 mM NaHCO_3_ pH 9.6) at 4°C for overnight. After washing three times with PBST buffer (Phosphate buffered saline containing 0.05% Tween-20, pH 7.4), the plates were blocked with 300 µL/well of 1% BSA in PBS buffer for 2 hat 37°C. The COE-mPIGS purified protein (10 μg/ml) were 2-fold serially diluted in PBS buffer containing 0.1% BSA and incubated at 37°C for 2 h. The mouse IgG antibody (Sigma) was used as the negative control. After washing three times with PBST buffer, the plates were incubated with 100 µl of anti-mouse IgG conjugated with alkaline phosphatase (1:7,000 dilution, Sigma A3562, St. Louis, MO, United States) as the secondary antibody, for 2 hat 37°C. Finally, 100 µl of phosphatase substrates (Sigma, S0942) was added per well for 15–20 min at room temperature and optical density measured at 405 nm in SUNRISE (Tecan, United Kingdom, CT) ELISA reader.

### Fcγ Receptor Binding Assay

Binding of antibody-antigen complexes to Fc receptors activates effector cells, triggering activities such as phagocytosis, inflammatory mediators release, and antibody-dependent cellular cytotoxicity (ADCC). Therefore, we tested the ability of binding of COE-mPIGS to Fc receptors present on J774 macrophage cells (ATCC), by flow cytometry. J774 cells (ATCC) were cultured in complete RPMI medium (2 mM L-glutamine, 10% Fetal Bovine Serum (FBS) in 5% CO_2_ incubator at 37 °C. The cells growing at 80% confluency were detached with dissociation buffer (Invitrogen), centrifuged at 1,000 rpm for 5 min and resuspend in binding buffer (PBS containing 3% BSA and 0.05% of sodium azide). 100 μg/ml of COE-mPIGS purified protein was added to 1 million cells in 100 μlcell suspension and incubated on ice for 2 h. Nonspecific binding was minimized by washing thrice with 3 ml of binding buffer. Cells were then incubated with 7.5 µl of anti-mouse IgG-FITC antiserum (the Binding Site) as the secondary antibody, for 1 h on ice. After washing, cells were resuspended in 500 µl of binding buffer and analyzed for green fluorescence on a Becton-Dickinson flow cytometer. Ten thousand cells were counted, and results expressed as the percentage of cells positive for FITC staining. Secondary antibody alone and unstained cells were used for background staining.

### Systemic Injection of Mice

All animal work was performed according to the requirements and guidelines of the Korean Animal Protection Act (APA) of 2008 by the Ministry of Agriculture, Food, and Rural Affairs, and the Laboratory Animal Act (LAA) of 2009 by the Ministry of Food and Drug Safety, Republic of Korea. The Five-week-old female BALB/c mice were purchased from Dong Yang Oriental Company (Korea) and randomly distributed into four groups for two independent immunization experiments. Mice were allowed at least 1 week to acclimate before start of vaccination. To test antigen immunogenicity, subcutaneous (s.c.) injection at the scruff of the neck under isoflurane anaesthesia was performed three times, at 3 weeks intervals, as described in Figure 4A. The mice were subcutaneously administered with 10 µg of bacterial COE (LSP removed) or 10 µg CoE-mPIGS protein, with or without 5 µg cholera toxin-CT (Sigma, C8052). Antigen combined with a commercial CT (Sigma) adjuvant or just PBS were used for comparison purposes. To analyze antigen-specific antibody responses from the mice sera, the blood was collected at weeks 3, 6, and 8 (end of the experiment) and sera prepared as described in Jespersgaard et al. (Jespersgaard et al., 1999). At the end of experiment, splenocytes were isolated to quantitate numbers of IgG-secreting cells (SCs) by ELISPOT or to perform lymphocyte proliferation assays as described previously (Kim et al., 2016).

### Oral Immunization of Mice

To test for oral immunogenicity, dried plant leaf powder from glyco-engineered uninfiltrated ΔXT/FT plants or agroinfiltrated plants containing 100 μg of COE-mPIGS, was re-suspended in 1 ml PBS buffer and weekly fed to five mice in each group by gavage needle (Figure 5A), for 6 weeks in total. The material expressing COE-mPIGS was used with or without Saponin Quillaja sp adjuvant (2.5 mg/mouse, Sigma, S4521) and bacterial recombinant COE protein alone and PBS buffer were used as control groups in the study. Mice were starved for 8 h before oral gavage for improved retention of the administered material. Blood and fecal samples were collected before immunization and 3 days after feeding at weeks 4 and 5, as previously described (Huy et al., 2016). Sera were recovered from coagulated blood by centrifugation at 13,200 rpm for 10 min and stored at −70 C, while fecal samples (approximately 200 mg) were suspended in 1 ml of PBS buffer supplemented with 0.01% sodium azide, to inhibit action of bacteria. The fecal extracts were collected by centrifugation at 13,200 rpm for 25 min and used for detection of sIgA antibody responses.

### Detection of Antigen Specific Antibody Responses

The ELISA was used to determine the specific serum antibody responses in immunized mice. The ELISA plates (96-well) were coated with 10 μg/μl of bacterial antigen (bCOE) in coating buffer and incubated at 37 C overnight. The plates were then washed thrice with PBST buffer (PBS buffer with 0.05% Tween-20) and blocked with 300 µl/well of PBS buffer containing 1% BSA at 37 C, for 2 h. Subsequently, the sera (starting dilution 1:100) and fecal extracts (starting dilution 1:4) were 2-fold serially diluted (in PBS buffer containing 0.1% BSA), and following 2 h incubation at 37 C, washed thrice with PBST buffer. 100 µl per well of 1:7,000 diluted anti-mouse IgG (Sigma) or anti-mouse IgA (Sigma) conjugated to alkaline phosphatase was then added and incubated for further 2 h at 37°C, followed by washing thrice with PBST buffer. For colour development, 100 µl/well of alkaline phosphatase *p*-nitrophenyl phosphate substrate system (Sigma, S0942-100 TAB) in 10% diethanolamine, 0.1% MgCl_2_, 0.02% sodium azide, pH 9.8, was added and plates incubated in the dark for 20 min, at room temperature. The optical density was measured at 405 nm using the SUNRISE reader (Tecan, United Kingdom), and endpoint titres were determined as 2xfold about background.

### ELISPOT Analysis

The Enzyme-Linked ImmunoSpot assay (ELISpot) was conducted to quantify antigen-specific antibody-secreting B cells (ASC). The PVDF membranes of ELISpot plate were activated by incubating in 35% ethanol for 10 min at room temperature. The plate was washed thrice with PBS buffer to remove residue ethanol, and 500 ng/well of bacterial antigen (bCOE) added, in PBS buffer at 4°C overnight. Following washing, 200 µl of complete RPMI containing 10% FBS was added and plates incubated at 37°C, 5% CO_2,_ for 1 h, to prevent non-specific binding. Splenocytes were isolated from mice 10 days after final immunization using Percoll density gradients. Viable splenocytes were counted by trypan blue method (Thermo Fisher Scientific). Cells were diluted in complete RPMI medium to 1 × 10^6^, 5 × 10^5^, and 1 × 10^5^ cells/well and then cultured at 37 °C, 5% CO_2_ for 2 days. After removal of unbound cells by washing, anti-mouse IgG alkaline phosphatase (Sigma) was added and following 2 h incubation at 37°C, the cells were washed three times as before. ASC were visualized by addition of BCIP/NBT phosphatase substrate (Sigma) and colour spots counted under microscope and computer.

### T-Cell Proliferation Assay

T-cell proliferation ability of splenocytes from immunized mice was determined by the [^3^H] thymidine incorporation method. Mononuclear cells (MNCs) were isolated from the spleens of immunized mice at 10-days after the final immunization. Splenocytes suspensions were filtered using 70-µm cell strainer and separated by percoll 40–70% gradient. The cells were washed three times with PBS buffer (Sigma-Aldrich) and re-suspended in complete RPMI medium containing 10% FBS (FBS; Invitrogen), 1 M HEPES, 1 M sodium pyruvate, 2 mM mercaptoethanol, 100 IU/ml penicillin and 100 μg/ml streptomycin. The cells were seeded in 96-well plates at 1 × 10^6^, 5 × 10^5^ and 1 × 10^5^ in 100 µL of complete RPMI medium, and 10 µg of bCOE added (or medium alone as the negative control). After 48 h incubation at 37°C in incubator 5% CO_2_, cells were pulsed with 0.5 µCi of [^3^H]-thymidine deoxyribose (TdR) (Amersham Life Science, Buckinghamshire, United Kingdom) per well, for an additional 18 h. Using a 96-well cell harvester (Inotech, Dottikon, Switzerland), the cells were collected and assessed for tritium incorporation using a liquid scintillation counter (Packard Instrument, Meriden, CT, United States). Stimulation indices were calculated by dividing the tritium incorporation (counts per minute) in cells treated with cognate antigens by the incorporation in control cells treated with PBS.

### Data Analysis

Representative data from at least two or multiple experiments are expressed as the means of replicate tests ±SD. The statistically significant differences were determined by one-way ANOVA, followed by Tukey’s for group-to-group comparisons, with values of *p* < 0.05 considered statistically significant. Statistical analysis was performed using IPMS^®^ SPSS^®^ statistics package, version 22.

## Results

### Gene Construction and Agro-Infiltration

**FIGURE 1:**
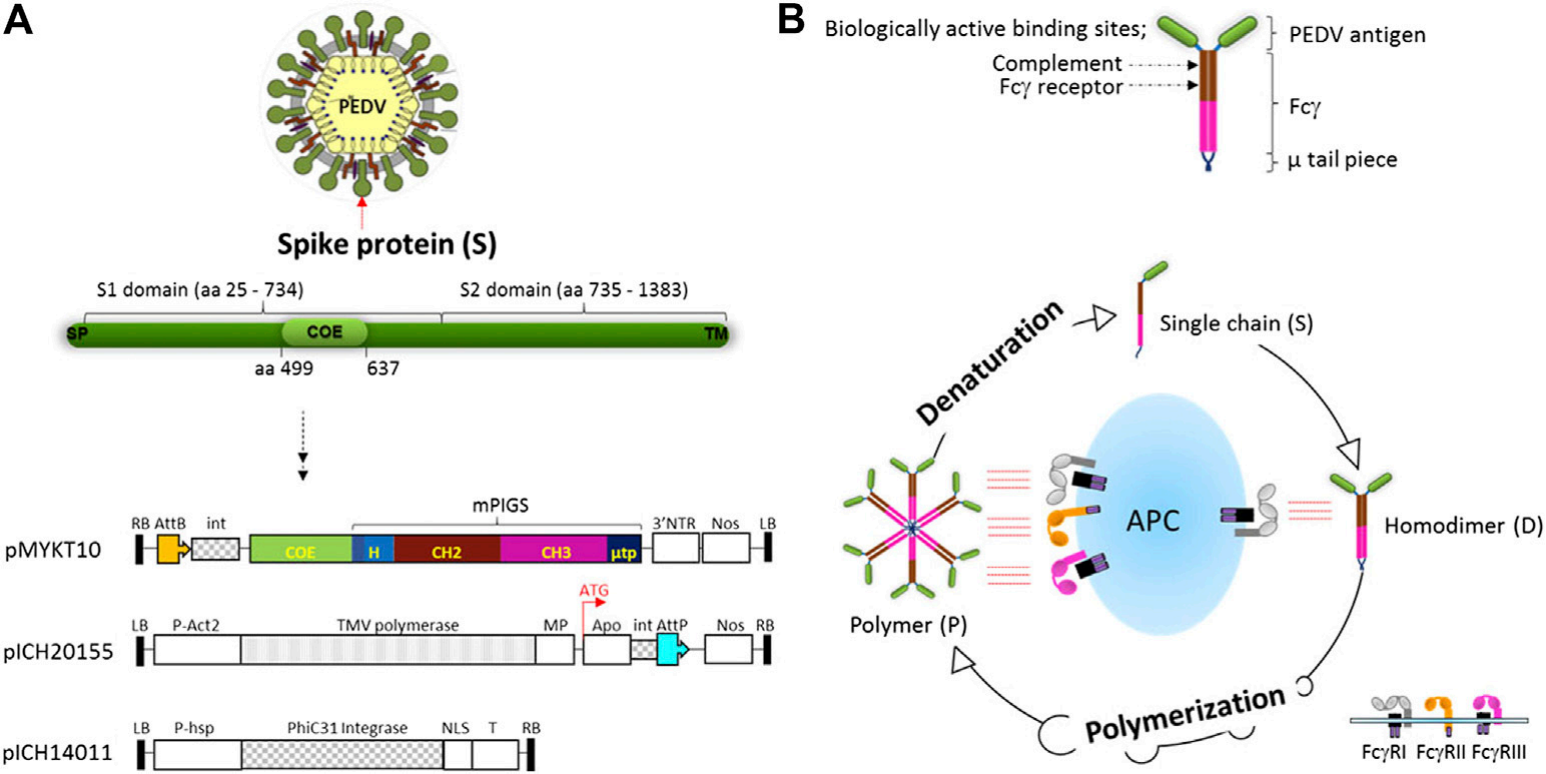
Vector construction for plant expression of COE-mPIGS and a schematic diagram of poly-Fc platform **(A)** the structure of S protein of PEDV and plant expression vector: three vectors were used to produce COE-PIGS simultaneously. pMYKT10, 3′ pro-vector containing COE-mPIGS gene; pICH20155, 5′ pro-vector containing apoplast signal peptide sequence; and pICH14011 vector for expression of the integrase. LB and RB, left border and right border of T-DNA; P-Act2, Arabidopsis actin 2 promoter; P-hsp, Arabidopsis heat shock protein hsp 81.1 promoter; NOS, nos terminator; 3′NTR, 3′ nontranslated regions of TMV; MP, movement protein; Apo, apoplast targeting pre-sequence; int, intron; AttP and AttB, recombination sites; NLS, nuclear localization signal; T, terminator. **(B)** Homodimeric structure of COE-PIGS presenting biologically active sites. COE, antigen of PEDV; Fcγ, Fc gamma chain based on mouse IgG2a; µ tp, µ tail piece of human IgM; PIGS, Polymeric IgG Scaffold. The process of polymerization from a single chain is shown in a lower diagram. Polymers bind to both high and low affinity Fc receptors (FcγI, FcγII, and FcγIII) embedded in antigen presenting cell (APC) resulting in enhancement of the immune responses, while monomeric IgG binds to only high affinity Fc receptor (FcγI). Polymers can be denatured into single chain under reducing conditions.

A causative pathogen of PED, coronavirus-like agent (CV777) was discovered by Belgium scientists in 1978 (Lee, 2015). Although the current vaccine based on the CV777 strain shows only partial protection, we took the epitope from this strain primarily to test our vaccine platform as a proof-of-concept, that immunogenicity can be enhanced by molecular means. The neutralizing epitope COE sequence that corresponds to the 15 kDa portion of the S protein was genetically fused to poly-Fc gene construct from mouse IgG (mPIGS) (Kim et al., 2017b). Both antigen and mPIGS genes were synthesized with high frequency of codon usage in plants. To produce recombinant PIGS fusion protein, pro-vector which was developed by Icon Genetics, was employed as the plant expression vector (Gleba et al., 2005). The linear DNA of COE-mPIGS gene inserted into Bsa I restriction enzyme site within 3′ pro-vector was used to express the construct transiently in tobacco cells. Tobacco leaves were agro-infiltrated by combining the expression vector with a 5’ pro-vector for apoplast targeting of protein in the cells, and the vector for expression of integrase (Figure 1A). All through the expression, LBA4404 agrobacterium harboring plasmid encoding J chain, which links together monomeric units in secretory IgA (SIgA) or pentameric IgM in nature (Yoo et al., 1999), was also co-infiltrated, to improve multimerization by forming a disulfide bridge with the mu-tail piece at the C-terminal end of mPIGS (Davis and Shulman, 1989; Frutiger et al., 1992; Fazel et al., 1997). The single chain of COE-mPIGS may be assembled as a homodimer or multimers, like a natural IgM antibody, leading to enhanced Fc-receptor mediated functions (Figure 1B). For expression, ΔXT/FT glyco-engineered tobacco plants (Strasser et al., 2008) were used to produce glycan-optimized COE-mPIGS.

### Expression of COE-mPIGS in *N.benthamiana* Leaves

**FIGURE 2:**
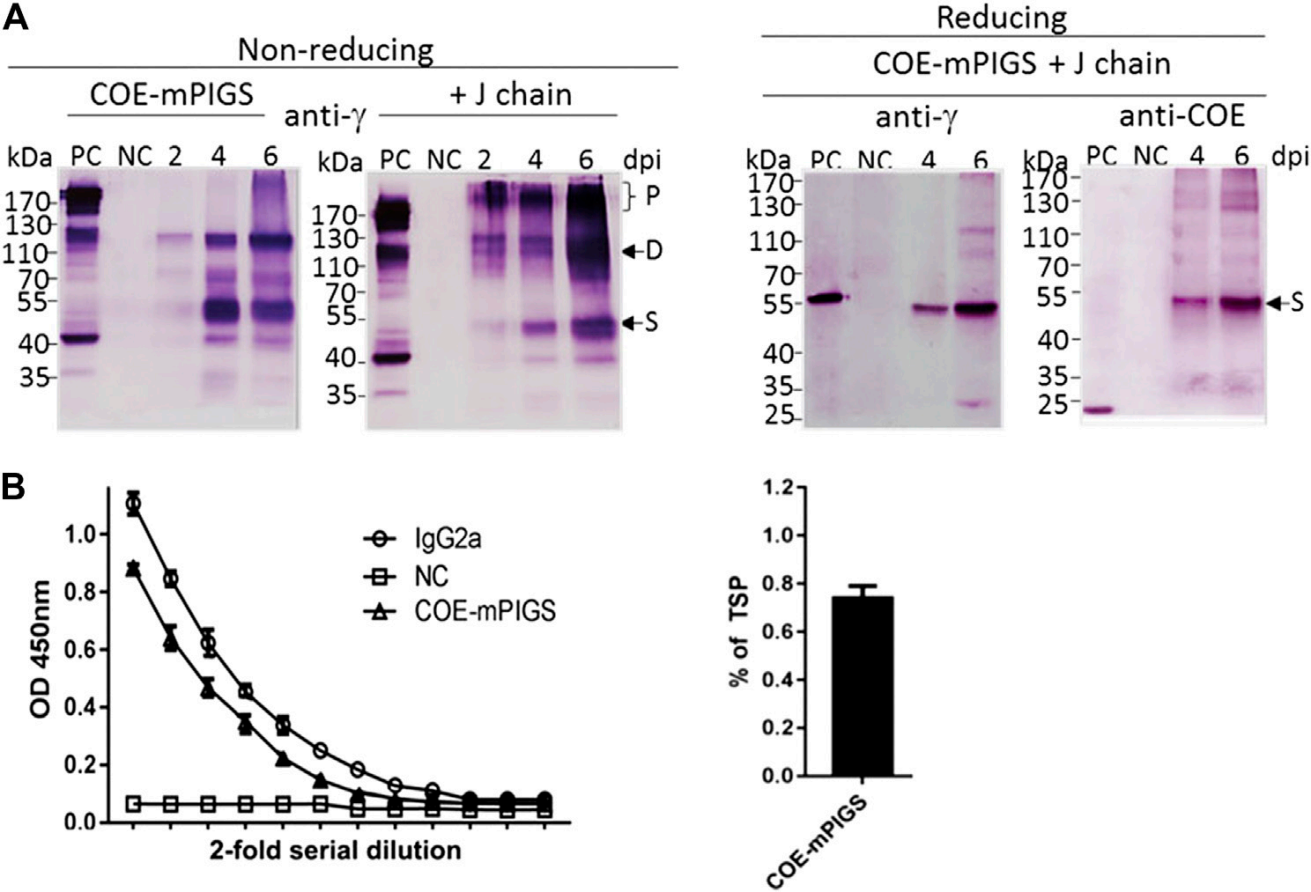
Expression of COE-mPIGS protein in tobacco **(A)** Immunoblot analysis of COE-PIGS co-expressed without/with J chain: the protein extracts from infiltrated and non-infiltrated leaf tissues were analyzed by probing with anti-mouse IgG gamma chain specific or anti-COE polyclonal antibodies under non-reducing and reducing conditions. PC, commercial mouse IgG or COE recombinant protein produced in *E. coli* used as positive controls; NC, non-infiltrated protein extract used as the negative control; lanes 2, 4, and 6, protein extracts from the harvested leaves at 2, 4, and 6 days post-infiltration (dpi). Molecular weight markers are shown on the left. **(B)** Indirect ELISA probing by anti-mouse IgG gamma chain specific antibody. The level of expression (approximately 0.7% of total soluble protein) was estimated by comparing to commercial IgG protein by ELISA.

For the protein assay, the tobacco leaves infiltrated with *A. tumefaciens* harboring three pro-vector components, with or without J chain construct, were extracted in PBS buffer and analyzed. Immunoblot analysis was conducted to detect the COE and mPIGS portions in protein extracts using anti-COE polyclonal sera or anti-mouse IgG heavy chain specific antibodies, respectively. Samples of 40 micrograms of protein were extracted at 2, 4, and 6 days post infiltration (dpi) and boiled before separating on SDS-PAGE gel. Following transfer, immunoblot membranes were probed by anti-IgG antibody under non-reducing conditions, showing varying sizes of COE-mPIGS, ranging from approximately 50–53, 100 kDa and above 170 kDa, possibly representing the single chains (S), homodimers (D) and polymers (P) (Figure 2A). There were significantly more polymeric forms in samples from 6 dpi compared to 2 or 4 dpi, and importantly, with co-expression with J chain compared to without J chain (Figure 2A left two panels). It was not possible to determine the exact molecular weight of the polymeric fraction or its proportion in the preparation, but as Figure 2A indicates, the inclusion of J chain markedly enhanced the proportion of dimers and especially polymers, suggesting their higher efficiency assembly, into likely pentamers of approximately 500 kDa, while in the absence of J chain the polymers likely represent hexamers of approximately 600 kDa. Under reducing conditions, COE-mPIGS fusion protein was dissociated into a single chain with molecular weight of approximately 53 kDa, as detected by anti-mouse IgG antibody and anti-COE polyclonal sera (Figure 2A right two panels). The single chain of COE-mPIGS calculated by ProtParam tools (https://web.expasy.org/protparam/) is 44.3 kDa, but that excludes 4 potential N-glycans. There was no cross-reactivity in protein extracts from non-infiltrated ΔXT/FT *N. benthamiana* plants used as a negative control. The recombinant COE purified from bacteria (bCOE) and a commercial IgG antibody were used as positive controls, yielding 15 and 55 kDa protein bands under reducing conditions, respectively (Figure 2A right two panels).

The amount of COE-mPIGS protein in the leaf tissues was measured using quantitative sandwich ELISA (Figure 2B left). Known amounts of IgG2a were used to generate the standard curve. The expression level of COE-mPIGS was approximately 0.74% of total soluble protein (Figure 2B right). There was no non-specific antibody binding in protein extracts from non-infiltrated leaves used as a negative control.

### Protein Purification

**FIGURE 3:**
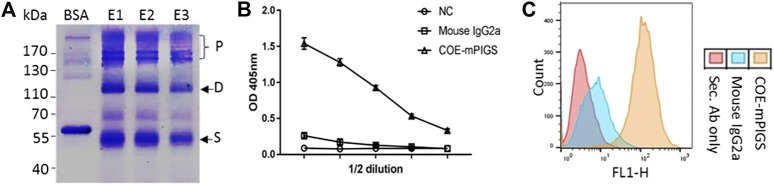
Purification of protein and a functional characterization assay **(A)** Recombinant COE-mPIGS was purified from plant extracts using protein A/G affinity chromatography methods. The proteins were separated by SDS-PAGE under non-reducing conditions and visualized by Coomassie brilliant blue staining. Lane BSA, bovine serum albumin; lane E1-E3: fractions of eluted protein. Pre-stained protein molecular weight standards and polymers (P), homodimer (D), and single chain (S) shown on the left and right sides of the figure. **(B)** C1q complement binding by ELISA shown as 2-fold serial dilutions of COE-mPIGS (5 μg/ml). The protein extracts from wild-type plant were used as the negative control. **(C)** Fc gamma receptor binding in J774 cells by FACS analysis. The COE-mPIGS binding histogram was overlaid to that obtained for mouse IgG2a antibody or secondary antibody alone, used as negative controls.

For protein purification, the complex COE-mPIGS with J chain was transiently expressed in ΔXT/FT *N. benthamiana* for 6 days and the leaves were collected. The recombinant COE-mPIGS protein was purified from protein extracts using protein A/G affinity chromatography method and the eluted fractions (E1-E3) were then analyzed by SDS-PAGE. As shown in Figure 3A, purification products were shown to be approximately 53 kDa (single, S), 110 kDa (dimer, D) and above 170 kDa (polymers, P) protein bands. The commercial BSA (Sigma) was used as protein standard for molecular weight comparison.

### Characterization of Functional COE-mPIGS Protein

Antigen-antibody complex (immune complex)-mediated activation of the complement system, and IgG Fc receptors (FcɤRs) mediated uptake, are important defence mechanisms mediated by innate and adaptive immune responses. Thus, the recombinant COE-mPIGS were tested for binding to C1q complement component and to Fc gamma receptor-bearing antigen presenting cells (APCs). As shown in Figure 3B, the COE-mPIGS bound to C1q in dose-dependent manner confirming the presence of polymeric forms of COE-mPIGS. Similarly, FcɤR targeting by COE-mPIGS revealed a significantly increased level of binding in mouse J774 macrophages, compared to secondary mouse IgG-FITC antibody only. In contrast, the monomeric IgG used as an internal control showed much less binding to both C1q and FcɤR (Figures 3B,C).

### Systemic and Oral Immunogenicity of COE-mPIGS

**FIGURE 4:**
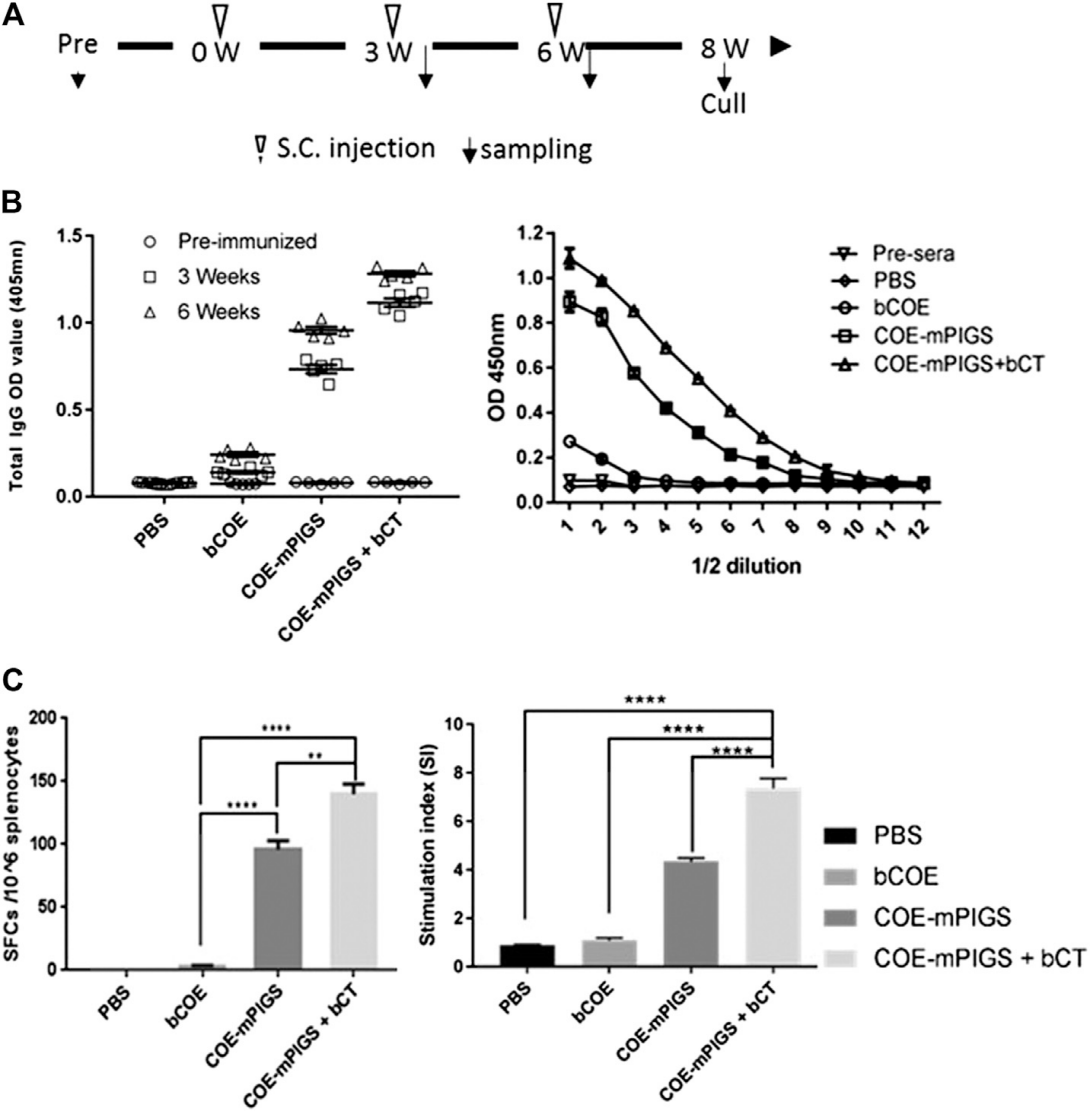
Mouse immune responses induced by systemic injection **(A)** Schedule for immunization and sample collection. The BALB/c mice were randomly distributed into four groups. Mice were subcutaneously injected with PBS only, or with 10 µg of the purified COE-mPIGS protein alone or with 5 µg of bacterial cholera toxin (bCT) as adjuvant. The blood samples were collected at 3 and 6 weeks post-immunization. **(B)** Detection of COE-specific serum IgG. COE-mPIGS and COE-mPIGS + bCT significantly enhanced IgG antibody responses after boosting, while COE and PBS induced only a low level or no response, respectively. The endpoint antibody titers were detected at approximately 1:51,200 and 1:204,800 for COE-mPIGS alone and COE-mPIGS with bCT, respectively. The results were shown as the mean of triplicate measurements ±SD of the 1:100 dilution **(left panel)**, and serial 2-fold dilutions starting from 1:100 dilution **(right panel)**. **(C)** Cellular immune responses. Spot forming units after bacterial COE antigen stimulation in pooled (n = 4) cultures were detected by ELISpot assay **(left panel)** and the results shown as the mean values of the number of antibody-secreting cells from triplicate cultures. bCOE protein induced only low-frequency antibody-secreting cells, while COE-mPIGS and COE-mPIGS adjuvanted with bCT induced high-levels of the antibody-secreting cells. T cell proliferation was assessed by [3H] thymidine incorporation assay **(right panel)**. Stimulation index was calculated by dividing the CMP (counts per minute) of Ag-stimulated cultures by CPM of control cultures from three separate experiments. **p < 0.01; ****p < 0.0005, by Tukey’s test.

To test for systemic immune responses, mice were subcutaneously immunized with bCOE or PBS buffer or COE-mPIGS with or without adjuvant (bacterial CT), on three separate occasions (Figure 4A). The serum IgG antibody immune responses were analyzed after weeks 3 and 6 by ELISA. As shown in Figure 4B, mice given only bacterial antigen bCOE showed low IgG antibody response, while mice immunized with COE-mPIGS with or without adjuvant showed high-levels of IgG. The endpoint antibody titers were detected at approximately 1:51,200 and 1:204,800 in groups immunized with COE-mPIGS alone and COE-mPIGS with bCT, respectively (Figure 4B). As expected, there were no anti-COE IgG responses in mice which received PBS buffer only, as the control. Furthermore, COE-mPIGS induced higher frequency of antibody secreting B cells, compared to antigen alone (Figure 4C, left), and this was further enhanced by the presence of the CT adjuvant. Similarly, we observed stronger antigen-specific T cell proliferation of splenocytes from mice immunized with COE-mPIGS compared to antigen alone, and like for B cells, this was further enhanced by the adjuvant (Figure 4C, right).

**FIGURE 5:**
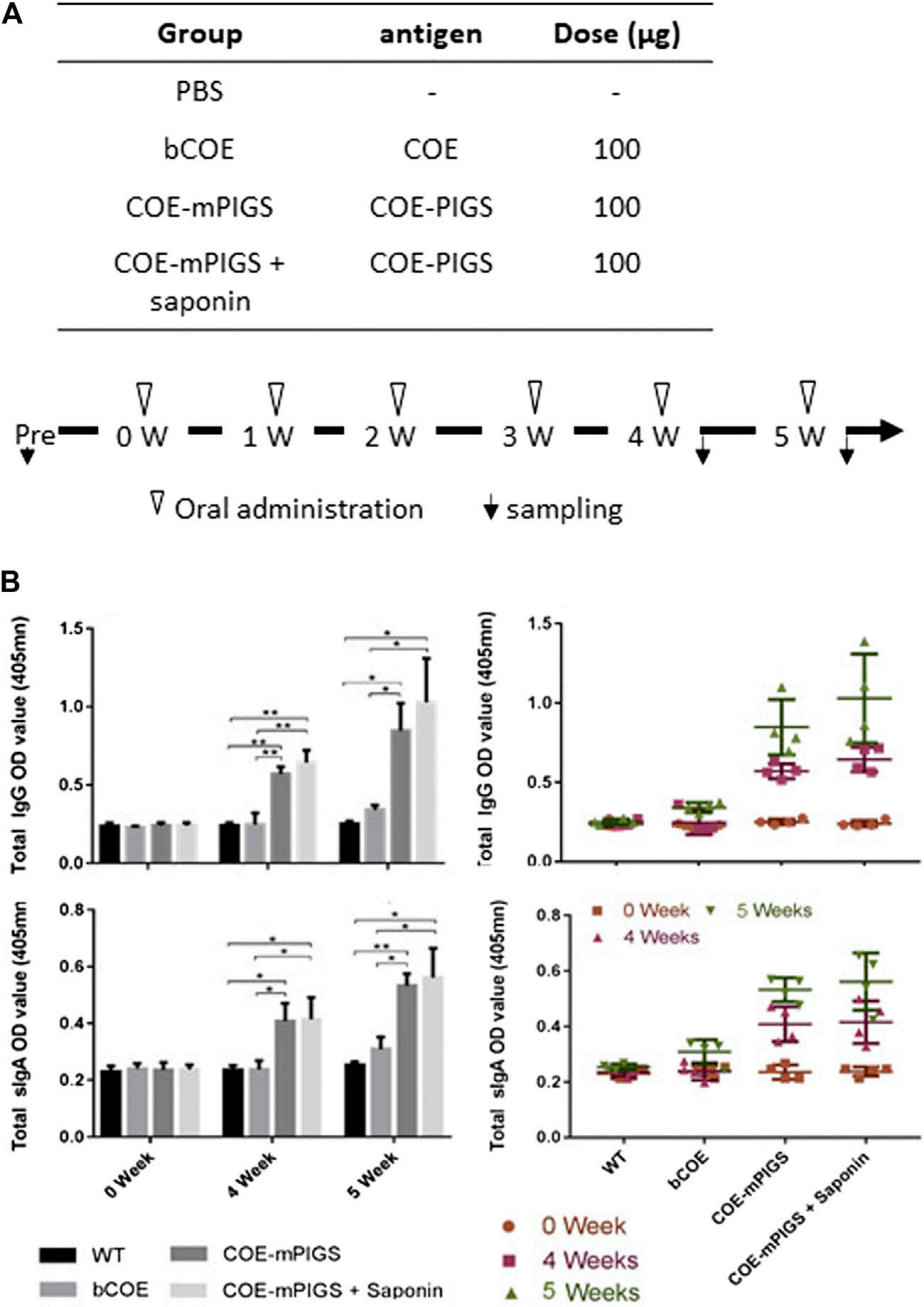
Mouse immune responses induced by oral administration **(A)** Protocol for immunization and sampling **(B)** Detection of COE-specific serum IgG **(top two panels)** and fecal IgA **(bottom two panels)** in pooled samples for each group **(left panels)** and the individual mice **(right panels)**, in 1:100 diluted serum and 1:4 diluted fecal extracts. COE-mPIGS and COE-mPIGS + Saponin induced significant IgG and IgA antibody titres after boosting. Only background responses were detected for COE and PBS. The results are shown as the mean of triplicate measurements ±SD. *p < 0.05, **p < 0.01 and ***p < 0.001, by Tukey’s test.

To test for oral immunogenicity, sera and fecal extracts were obtained from mice immunized with PBS, bCOE, and COE-mPIGS alone or COE-mPIGS with tor without he saponin adjuvant, and analyzed for COE specific IgG and IgA antibodies. Saponin was used in this case as it is generally considered to be a good oral adjuvant (Cox et al., 1998; Lee et al., 2015; Pickering et al., 2006; Rajput et al., 2007; Sjolander and Cox, 1998; Sun et al., 2009). Both serum IgG and fecal IgA against COE were detected in mice immunized with COE-mPIGS alone and when combined with saponin, at 4 and 5 weeks (Figure 5B). While endpoint titres were not determined for fecal IgA, based on single dilution measurement and internal ELISA normalization, they could be inferred to be significantly lower that serum IgG, at an estimated 1:1,000–1:2,000. Interestingly, COE-mPIGS with saponin induced only marginally enhanced serum IgG and fecal IgA antibody responses, but this was not statistically different to COE-mPIGS alone. While this was surprising, similar results were also reported in another study of the oral immunogenicity of the tEDIII-Co1 dengue antigen expressed in rice calli (Kim and Kim, 2019), suggesting that in our hands at least, saponin did not exert strong gut adjuvanticity. However, taken together, our data show that COE-mPIGS alone could induce both systemic and mucosal antigen specific immune responses, and that combining these routes of delivery could be used to effectively immunize against a gut pathogen like PEDV.

## Discussion

In this study, we demonstrated enhanced immunogenicity of an antigen against PEDV by molecular engineering of adjuvanticity. The neutralizing epitope derived from CV777 strain, COE, was fused to the polymeric Fc scaffold of IgG, as a new and promising vaccine platform for induction of both systemic and mucosal immune responses via the oral route. Our choice of antigen was the COE epitope containing sequence, which was demonstrated recently to induce PEDV neutralizing antibodies in mice when expressed in plants in native trimeric form (Ho et al., 2020). However, we emphasize that our primary aim was to develop a suitable vaccine platform against this and similar veterinary pathogens, rather than a ready-made vaccine, as that would require further modifications, including inclusion of other portions of the S protein as well as different variants of the virus.

PEDV belongs to the family *Coronaviridae*, genus *Alphacoronavirus* relating to transmissible gastroenteritis virus or transmissible gastroenteritis coronavirus (TGEV). There is no cross-protection between TGE and PEDV even though both are Coronaviruses and show similar clinical signs. It only infects pigs and does not relate to human diseases caused by beta-coronavirus such as Severe Acute Respiratory Syndrome (SARS), Middle East Respiratory Syndrome (MERS) and Severe acute respiratory syndrome coronavirus 2 (SARS-CoV2 or COVID-19). PEDV is one of the major causes of high morbidity and mortality in piglets worldwide and currently, inactivated and live attenuated vaccines have been extensively used, but with limited success only. To compound this, the high frequency of recombination events in coronaviruses may result in the generation of novel viruses with high genetic diversity, which could invoke unpredictable changes in virulence during infections (Zhao et al., 2017; Ou et al., 2020). Thus, the emergence of highly virulent strains and the recurrent outbreaks even in vaccinated farms highlight the need for more effective vaccines. Recently, Park et al., demonstrated that the newly isolated PEDV strain conferred critical passive immune protection to pigs against epidemic PEDV infection (Park et al., 2018). An alternative form of immunization against PEDV might be a mucosal vaccine, which could control the infection at the sites of pathogen replication in porcine small intestinal villous epithelial cells or enterocytes (Lee, 2015). Yuchen Li et al. demonstrated that PEDV could cause typical diarrhea in piglets through a nasal spray (Li et al., 2018). That provides evidence for possible airborne transmission and subsequent gastrointestinal dissemination and is useful in facilitating strategies for PEDV prevention.

Generally, the S protein of coronavirus consists of S1 and S2 domains which mediate attachment and membrane fusion, respectively. The N-terminal region of the S protein, S1 domain (amino acid position 231-733 in CV777, NCBI assess no. NP 598310.1; 234-736 in Korean isolate, 2005, NCBI assess no. AAM19716.1; 235-737 in 85-7 mutant, NCBI assess no. AST13221.1; 234-736 in HUA-PED96 Vietnam isolate, NCBI assess no. ASU09581.1; 230-732 in Chinese isolate, 2013, NCBI assess no. AGN29320.1), is important for recognizing the Porcine aminopeptidase N (pAPN) receptor, which is predominantly expressed on the surface of epithelial cells of small intestine, and has been identified as the cellular receptor for PEDV (Nam and Lee, 2010). The summary of S protein features of PEDV published in NCBI and UniProt was included in Table 1. Many of subunit vaccine candidates against coronaviruses have been developed using S and S1 domain, or host cell receptor binding domain such as ACE2 (aa 321-536, NCBI assess no. MN908947, NC_045512, MN985325, MN988669) against SARS or SARS-CoV2 (Chakraborti et al., 2005; He et al., 2004; He et al., 2006; Ou et al., 2020). Thus, vaccine strategy targeting to pAPN receptor binding domain could be used to protect against PEDV even though S glycoprotein of PEDV is not cleaved into S1 and S2, in contrast to beta- and gammacoronaviruses split by furin enzyme located between the two domains (Ou et al., 2020). An alternative antigen to induce neutralizing antibodies suggested by Chang et al., is called S1D fragment (residues 636–789) in CV777 strain, located between S1 and S2 domain, and consisting of two linear epitopes. Anti-S1D5 (residues 744–759) and S1D6 (residues 756–771) antibodies reacted with the native S protein of PEDV. Furthermore, two core epitopes on S1D5 (^748^YSNIGVCK^755^) and S1D6 (^764^LQDGQVKI^771^) were detected by Pepscan analysis in a previous study (Sun et al., 2008). More recent studies, however, identified several neutralizing monoclonal antibodies all binding to the S2 domain of the spike protein (Okda et al., 2017). In fact, in that study, the S2 specific monoclonal antibodies were also more neutralizing than those targeting the S1 domain. This is interesting as it may suggest that the S2 domain should be included in the antigen moiety of any new PEDV vaccine, though it is not clear if natural infection also induces predominantly S2 targeted antibodies. This is not uncommon for viruses as for example, there are many strongly neutralizing antibodies against the domain three of the dengue E glycoprotein (Crill and Roehrig, 2001; Shrestha et al., 2010) and yet, natural infection does not induce such antibodies (Wahala et al., 2009; Williams et al., 2012).

In this study, we employed poly-Fc of IgG as an antigen delivery platform, with enhanced avidity of binding to both high and low affinity Fc gamma receptors (Kim et al., 2017b; Kim et al., 2018; Webster et al., 2018). This is an improvement on IgM which despite being polymeric (pentamer or hexamer naturally) displays low binding affinity for its receptor (Vollmers and Brandlein, 2006), or monomeric IgG which binds to only high affinity Fc gamma receptor. This feature of poly Fc fusion as a vaccine is expected to increase the efficiency of antigen-presentation compared to uptake of a single antigen by the immune cells, as schematically shown in Figure 1B. Also, binding to complement component (C1q) in an antigen-antibody immune complex manner could further enhance the adjuvant effect by activating complement cascade *in vivo* (Dempsey et al., 1996). In our previous studies, those functions were demonstrated using antigens of human diseases such as dengue (Kim et al., 2017b; Kim et al., 2018) and tuberculosis (TB) (Webster et al., 2018). Although the initial attempts at generating Fc fusions showed only partial success (Mekhaiel et al., 2011), further modifications and improvements of the original molecule suggested by Smith et al. (1995), as described for our polymeric platform, have enabled the application of this concept as an efficient vaccine delivery system.

Here we utilized the poly-Fc technology to develop a swine PEDV vaccine platform through either systemic injection or mucosal (oral) administration, which is relevant and potentially applicable to many enteric or respiratory diseases, such as TGEV, ASFV, Influenza A Virus in Swine (IAV-S), porcine reproductive and respiratory syndrome (PRRSV) and foot-and-mouth (FMDV) caused by *Coronaviridae*, *Asfarviridae, Orthomyxoviridae, Togaviridae* and *Picornaviridae* virus, respectively. These diseases are a significant economic burden in pork industry worldwide, and in need of better control through vaccination. Our results showed that both antigen-specific humoral antibody and cellular responses could be induced by either subcutaneous injection (Figure 4) or oral delivery (Figure 5) of COE-PIGS. Furthermore, in our other experiments (data not shown), the recombinant S1D fusion PIGS (S1D-PIGS) protein administrated to mice intranasally induced robust anti-S1D IgG and IgA antibodies in sera and bronchoalveolar lavage and a balanced Th1/Th2 cellular responses in the spleen, thus further strengthening the evidence for the utility of the mucosal route. As a further proof of evidence for this, we previously demonstrated that crude extracts containing antigens expressed in rice cells, including fragment ApxII from *Actinobacillus pleuropneumonia* and COE–Co1 fusion protein for PEDV, when given to mice by intranasal or oral route, also elicited antigen-specific IgG and IgA in spleen and Peyer’s patches (Huy et al., 2012; Kim et al., 2017a). Likewise, transgenic rice whole cells expressing a dengue antigen fused to cholera toxin B subunit (CTB) or enterotoxigenic *Escherichia coli* Heat-Labile Toxin B Subunit, when delivered orally, also induced systemic and mucosal immune responses (Kim et al., 2010; Kim et al., 2016; Kim and Kim, 2019). Finally, another PEDV antigen (S1D), when fused to CTB and expressed in tobacco cells also induced serum IgG and fecal IgA responses against both CTB and S1D, following oral immunization (Huy et al., 2016). However, those mucosal responses were induced when antigen was fused to CTB (well known as a general/mucosal adjuvant) or Co1 ligand (M-cell target peptide of epithelial cells), while a proof of oral immunogenicity by PIGS fusion molecules alone has been demonstrated for the first time in this study.

The limitation of our study is that polymerization of COE-PIGS in plants cannot be controlled, although it can be significantly enhanced by inclusion of the J chain. IgM can form both hexamers and pentamers, with the latter being facilitated by the J chain. We indirectly conclude but provide no experimental evidence, that most of the generated COE-PIGS may also be pentameric. Functionally though, both pentamers and hexamers (and even dimers) would retain the capacity for improved binding to the FC-gamma receptors and enhanced antigen presentation. Another limitation of our study is that, while our primary aim was to validate the immunogenicity of this new vaccine platform, we were unable to perform virus antibody neutralization assays, or the *in vivo* pathogenic challenge studies, as the mouse is not a suitable model for PEDV. Likewise, we cannot infer that the antigen dosing in mice would be replicated in pigs, as these are much larger animals. However, unlike drug treatments, vaccine scale up in different-sized hosts does not follow the same principles, and the optimal dose for pigs would have to be established experimentally.

Going forward, and to address the above limitations, we now intend to test this vaccination concept in pigs (the target animal), by replacing the mouse Poly-Fc with that from swine and expressing it in plants. Further modifications will include inclusion of additional portions of the S protein. While this vaccine candidate can be expressed in one of the more conventional expression systems, such as mammalian cells (Kim et al., 2017b), we propose plants as this emerging biotechnology has shown considerable promise, with several plant-expressed pharmaceuticals in human clinical trials. Success examples of this technology are illustrated by the deployment of the ZMapp antibody during the 2014 Ebola epidemic (Lyon et al., 2014) and the phase II clinical trial of a quadrivalent seasonal influenza VLP vaccine by Medicago Inc. (Pillet et al., 2019). In particular, animal vaccine development may benefit the most from this technology, due to somewhat less stringent GMP requirements and the associated costs of production, with the attractive possibility of delivering the vaccine in edible form, through feeding.

## Data Availability

The original contributions presented in the study are included in the article/Supplementary Material, further inquiries can be directed to the corresponding author.
